# Frühkindliche psychische Störungen: Exzessives Schreien, Schlaf- und Fütterstörungen sowie Interventionen am Beispiel des „Münchner Modells“

**DOI:** 10.1007/s00103-023-03717-0

**Published:** 2023-07-04

**Authors:** Margret Ziegler, Ruth Wollwerth de Chuquisengo, Volker Mall, Maria Licata-Dandel

**Affiliations:** 1 Present Address: Technische Universität München, München, Deutschland; 2 Sozialpädiatrisches Zentrum und Klinik für Sozialpädiatrie, Kbo-Kinderzentrum München, Heiglhofstr. 65, 81377 München, Deutschland

**Keywords:** Exzessives Schreien, Schlafstörungen, Fütterstörungen, Eltern-Kind-Beziehung, Eltern-Säuglings‑/Kleinkind-Psychotherapie, Excessive crying, Sleeping disorders, Feeding disorders, Parent–child relationship, Parent–child psychotherapy

## Abstract

Bis zu 20 % aller gesunden Säuglinge und Kleinkinder zeigen in den ersten Lebensjahren psychische Störungen im Sinne von untröstbarem Schreien (sog. Schreibabys), Schlaf- und Fütterstörungen. Nach Frühgeburt und bei Kindern mit neuropädiatrischen Erkrankungen finden sich noch deutlich häufiger vor allem langanhaltende Fütterstörungen und Schlafstörungen. Langfristig können sich daraus internalisierende und externalisierende Störungen im späteren Kindesalter entwickeln, häufig ist die Eltern-Kind-Beziehung belastet. Die Eltern schildern schwere Erschöpfung, extreme Verunsicherung und Hilflosigkeit.

Kinderärztinnen und Hebammen sind die ersten Anlaufstellen für die Familien. Schreibabyambulanzen, wie die 1991 von Mechthild Papoušek gegründete „Münchner Sprechstunde für Schreibabys“ am kbo-Kinderzentrum-München, sind für die hochbelasteten Familien ein niederschwelliges Angebot und leisten einen wichtigen Beitrag zur Prävention von Vernachlässigungen, Misshandlungen und psychischen Folgeerkrankungen des Kindes. Behandlungskonzepte basieren auf der Eltern-Kleinkind- und Bindungsforschung und integrieren kind- und elternbezogene Therapieansätze.

Während der COVID-19-Pandemie sind die psychosozialen Belastungen in den Familien gestiegen; dies war auch in den Schreibabyambulanzen deutlich spürbar.

## Abstract (en)

About 20% of all healthy infants and toddlers show problems in the area of mental health during their first years of life such as inconsolable crying (so-called cry-babies), sleeping problems, and feeding problems. The prevalence of enduring feeding problems and sleeping problems is distinctly higher in premature children and in children with neuropediatric disorders. These problems present a higher risk for the development of internalizing and externalizing disorders of mental health in later childhood. The parent–child relationship is often strained. Parents report experiencing severe exhaustion, extreme uncertainty, and helplessness.

Pediatricians and midwives are the first points of contact for families. Outpatient clinics for cry-babies such as the “Munich Consultation for Cry-Babies,” founded by Mechthild Papoušek in 1991 at the kbo-Children’s Center Munich, provide a low-threshold service for the highly stressed families. They can contribute to the prevention of neglect, maltreatment, and psychological secondary disorders of the child. Intervention strategies are based on parent–infant and attachment research and integrate child- and parent-oriented approaches.

During the COVID-19 pandemic, psychosocial stress factors in families increased. This development was also observable in the outpatient clinics for cry-babies.

## Einleitung

Als vor über 30 Jahren die „Münchner Sprechstunde für Schreibabys“ am kbo-Kinderzentrum München (Sozialpädiatrisches Zentrum, Institut für Sozialpädiatrie) eröffnet wurde, gab es geteilte Reaktionen. Kritische Stimmen meinten gar, es werden Bedürfnisse geweckt, die es eigentlich gar nicht gebe. Eltern und Kinderärzt:innen waren von Anfang an dankbar, ein offenes Ohr und Rat zu finden, in einer Phase von Überforderung und Hilflosigkeit [1]. Von Beginn an war es der Initiatorin und Gründerin Mechthild Papoušek ein Anliegen, neben der Erforschung dieser kindlichen Verhaltensauffälligkeiten und Belastungen der frühen Eltern-Kind-Beziehungen den hochbelasteten Familien eine Beratung und Therapie anbieten zu können [2]. Mehr als 10.000 Familien mit ihren Säuglingen und Kleinkindern haben sich seither an diese Spezialambulanz gewendet, nicht nur bei exzessivem Schreien in den ersten Lebensmonaten, den sogenannten Dreimonatskoliken, sondern auch bei Ein- und Durchschlafstörungen, Fütter- und Essverhaltensstörungen, exzessivem Trotzen oder Klammern und auch Hinweisen auf frühkindliche Bindungs- und Beziehungsstörungen. Institutionen verweisen auf Schreibabyambulanzen bei Gefahr der Kindesmisshandlung und Vernachlässigung. Deutschlandweit gibt es nun Beratungsstellen für Familien mit Säuglingen und Kleinkindern, viele arbeiten nach dem „Münchner Modell“.

Säuglinge und Kleinkinder erfahren in den ersten Lebensjahren eine rasante Entwicklung in allen Bereichen. Zu den psychoemotionalen Reifungs‑, Anpassungs- und Lernprozessen gehören der Aufbau von Bindungsbeziehungen zu den wichtigsten Bezugspersonen [3] und die Anpassung des Säuglings an seine Umwelt mit Selbst- und Verhaltensregulation, Regulation von Aufmerksamkeit, Emotionen und Affekt [4]. Die Säuglingsforschung der letzten Jahrzehnte hat eindrücklich beschrieben, dass Säuglinge und Kleinkinder aufgrund ihrer physiologischen Unreife unbedingt auf die co-regulatorische Unterstützung ihrer Bezugspersonen angewiesen sind, welche die spezifischen Bedürfnisse ihrer Kinder feinfühlig erkennen und sie in den phasenspezifischen Anpassungs- und Entwicklungsaufgaben unterstützen [5]. So helfen sie dem Baby, bei Schreien, Erregung und Irritationen wieder in einen ausgeglichenen Zustand zu kommen. Die Entwicklung eines Kindes kann somit niemals unabhängig vom familiären Umfeld betrachtet werden. Störungen der frühkindlichen Verhaltens- und Affektregulation sind von daher keine Störungen des Säuglings allein, sondern entstehen im Kontext einer entgleisenden Co-Regulation der gemeinsam zu bewältigenden Entwicklungsaufgaben [6]. Von daher werden in die Diagnostik und Behandlung von Säuglingen und Kleinkindern immer beide Elternteile einbezogen.

Exzessives Schreien, Schlaf- und Fütterstörungen sind die häufigsten psychischen Störungen im frühen Kindesalter. In diesem Beitrag wird auf die Klassifikation, Epidemiologie, das klinische Bild, die Ätiologie und den Verlauf dieser Störungen, der sogenannten Regulationsstörungen, eingegangen. Die Interventionen werden vorwiegend in Bezug auf das Münchner Modell einer integrativen Eltern-Säuglings‑/Kleinkind-Beratung und -Psychotherapie dargestellt.

## Klassifikation und Epidemiologie

„Unser Baby schreit Tag und Nacht; wir haben schon alles versucht, nichts hilft es zu beruhigen, wir sind so verzweifelt und hilflos, haben uns das alles ganz anders vorgestellt.“

Wann spricht man von einem Schreibaby, wie viel Schreien am Tag ist noch als normal bzw. erwartbar anzusehen? Ist dies als eine psychische Störung des Babys oder Extremausprägung einer normalen Entwicklung einzuordnen oder als belastendes Symptom mit Überforderung der Eltern?

**Figure 1:**
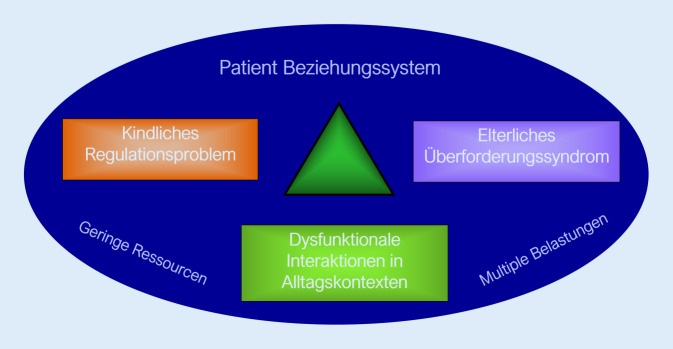

Im deutschsprachigen Raum hat sich bei frühkindlichen psychischen Auffälligkeiten der Begriff der frühkindlichen Regulationsstörung etabliert. In der Neuauflage der AWMF^1^-Leitlinie für Psychische Störungen im Säuglings- und Kleinkindalter wird dieser Störungsbegriff für multiple Regulationsprobleme in den Bereichen Schreien, Schlafen und Füttern aufgegriffen [7]. Nach Papoušek [6] wird unter der frühkindlichen Regulationsstörung eine Trias aus kindlicher Problematik der Affekt- und Verhaltensregulation, elterlicher Überforderung mit emotionaler Belastung und gestörten, dysfunktionalen Interaktionen verstanden (Abb. 1). Daniel Stern [8] benennt das „Beziehungssystem“ zwischen Eltern und Kind als den eigentlichen „Patienten“. Die Klassifikationen für psychische Störungen ICD^2^-10 und DSM^3^-5 sehen für das Säuglings- und Kleinkindalter kaum Diagnosen vor und schon gar nicht eine Diagnose für das Beziehungssystem zwischen Säugling (Patient:in) und Bezugspersonen. Die ICD-11 orientiert sich am DSM‑5 und sieht ebenso kaum spezifische Diagnosen für die ersten Lebensjahre vor.

Die DC:0–5^4^ ist ein eigenständiges, multiaxiales Klassifikationssystem für Säuglinge und Kleinkinder bis zum vollendeten 5. Lebensjahr [9]. Sie soll die psychische Störung des Kindes und Belastungen seiner sozialen Beziehungen und seiner Umwelt abbilden. Dazu stehen 5 Achsen zur Verfügung, die alle in eine Beurteilung des psychischen Befundes einfließen sollten:

- Achse I: klinische (psychische) Störungen in 10 Kategorien,
- Achse II: Beziehungskontext zwischen den Bezugspersonen und der versorgenden Umgebung mit dem Kind,
- Achse III: körperliche Gesundheit und Krankheiten,
- Achse IV: psychosoziale Stressoren,
- Achse V: Entwicklungskompetenzen.

Zu den Störungskriterien gehört, dass sich die Störung des Kindes deutlich negativ auf das Kind und/oder die Familie auswirkt und die Teilhabe des Kindes einschränkt. In der DC:0–5 finden sich „neue“, bisher nicht verwendete Störungen auf Achse I, die differenzierter die Situation des Kindes darstellen, z. B. die Diagnose einer Beziehungsstörung, die nicht nur das Kind, sondern auch die Bezugspersonen miteinschließt. Es sind die Diagnosen Schreistörung der frühen Kindheit, Schlafstörungen und Essstörungen spezifisch für das Säuglings- und Kleinkindalter formuliert.

Bis zu 20 % aller gesunden Säuglinge und Kleinkinder zeigen in den ersten Lebensjahren psychische Auffälligkeiten bzw. Störungen. Je nach Erhebungsinstrumenten und Falldefinition wird in den westlichen Industrieländern für das exzessive Schreien in den ersten Lebensmonaten eine Prävalenz zwischen 5 % und 26 % angegeben [10, 11]. Häufig wird dabei die sogenannte Dreierregel nach Wessel benannt: exzessives, nicht tröstbares Schreien von mehr als 3 h am Tag, mehr als 3 Tage pro Woche, während mehr als 3 aufeinanderfolgenden Wochen. Für die Ein- und Durchschlafstörungen wird nach Alter des Kindes differenziert. Nach DC:0–5 liegt eine Einschlafstörung ab dem Alter von 6 Monaten vor, wenn das Einschlafen länger als 30 min dauert und die Bezugspersonen involviert sind. Eine Durchschlafstörung kann ab dem Alter von 8 Monaten diagnostiziert werden, wenn das Kleinkind mehrfach oder länger in der Nacht erwacht und die Eltern zum Wiedereinschlafen einfordert [9]. Die Prävalenz für Ein- und Durchschlafstörungen liegt bei 10–33 % [11, 12]. Leichte bis mittelschwere Fütter- oder Essverhaltensstörungen mit und ohne Gedeihstörungen werden mit 15–25 % angegeben [11, 13, 14]. Bei Säuglingen und Kleinkindern mit chronischen Erkrankungen, Entwicklungsstörungen oder schweren neuropädiatrischen Erkrankungen wie Cerebralparese finden sich bei über 50 % der Kinder z. T. schwere Fütterstörungen [13].

## Klinisches Bild: exzessives Schreien, Schlafstörungen, Fütterstörungen

„… wir haben uns mittlerweile damit abgefunden, dass wir ein „high need baby“ haben, aber die Nächte mit stündlichem Aufwachen und Schreien halten wir nicht mehr lange durch …“

Exzessiv schreiende Babys schlafen im Vergleich zu anderen Babys deutlich weniger, sind schnell überreizt, kommen vor allem am Tag kaum zur Ruhe und zum Schlafen und sind auch schwer oder manchmal über Stunden nicht zu beruhigen [1, 15]. Die Eltern fühlen sich hilflos und überfordert, kommen an ihre Grenzen. Bei Verunsicherung und fehlender Unterstützung und Entlastung entstehen Teufelskreise aus Überforderung der Eltern mit Rückzug und nicht ausreichender Co-Regulation des Babys. Im klinischen Alltag ist der Belastungsgrad der Eltern ausschlaggebend, auch wenn die Kriterien für die Definition einer Störung nicht voll erfüllt sind. Vor allem bei exzessivem Schreien und kindlichen Schlafstörungen besteht das Risiko einer Kindesmisshandlung (z. B. Schütteltrauma) durch die überforderten Eltern.

Schlafstörungen im Säuglings- und Kleinkindalter zeichnen sich dadurch aus, dass das Kind z. T. nur mit sehr aufwendigen elterlichen Einschlafhilfen zur Ruhe und zum Schlafen kommt und auch bei nächtlichem Erwachen diese von den Eltern einfordert. Häufig folgen Schlafstörungen nach der Phase der Dreimonatskoliken. Die Kleinkinder zeigen weiterhin Probleme der Selbstregulation. Verunsicherungen der Eltern, was sie ihrem Kind im Schlafenkontext bereits zutrauen können, begünstigen die Schlafstörungen. Eltern, die Beratungsstellen aufsuchen, sind häufig hochgradig übermüdet und erschöpft, schildern häufig ambivalente Gefühle gegenüber ihrem Kind und sind nicht mehr alleine in der Lage die dysfunktionalen Interaktionen mit ihrem Kind zu verändern [2].

„Jede Mahlzeit ist ein Kampf, alles dreht sich nur ums Essen; zum Spielen bleibt keine Zeit und Energie …“

Fütter- und Essverhaltensstörungen des Kindes können, müssen aber nicht mit einer Gedeihstörung einhergehen [14]. Das Kind zeigt bei den Mahlzeiten Abwehr, scheinbar Appetitlosigkeit und Unlust zu essen; die Eltern reagieren mit immer neuen Essensangeboten, aber auch mit Druck und Zwang beim Füttern oder bieten ständig Essen an, auch Trinken im Halbschlaf oder beim Spielen [16]. Teufelskreise verfestigen sich mit Ängsten der Eltern um Überleben und Gedeihen und schließlich auch unzureichender oder einseitiger Nahrungsaufnahme des Kindes [13]. Bei Säuglingen und Kleinkindern mit organischen Erkrankungen und Entwicklungsstörungen können auch Beeinträchtigung in der Nahrungsaufnahme (z. B. mundmotorische Störungen, gastroenterologische Erkrankungen) vorliegen.

## Ätiologie

In Bezug auf spezifische Ursachen frühkindlicher Regulationsstörungen wird ein Zusammenwirken biologischer und psychosozialer Faktoren diskutiert. Jedoch gibt es bislang kaum Forschung, die bezüglich der Ätiologie ein biopsychosoziales Modell empirisch bestätigen würde, da zur Klärung dieser Frage Längsschnittstudien benötigt werden, die im Optimalfall bereits am Anfang (oder sogar vor) der Schwangerschaft beginnen.

Querschnittstudien weisen auf negative Zusammenhänge zwischen der Qualität der Eltern-Kind-Interaktion und Problemen in den Bereichen Füttern [17] und Schlafen [18] hin, wobei in Bezug auf exzessives Schreien keine konsistenten Zusammenhänge gefunden wurden [19]. Säuglinge mit Schwierigkeiten in der Selbstregulation können der Mutter gegenüber weniger zugewandt [20, 21] und besonders vulnerabel für unfeinfühliges Verhalten sein [22]. Jedoch lassen diese Befunde keine Rückschlüsse auf Kausalität zu. Bidirektionale Effekte zwischen kindlichen Regulationsproblemen und Eltern-Kind-Interaktionsqualität erscheinen wahrscheinlich: Durch stark ausgeprägte Regulationsprobleme auf Kindseite kann es zu Einschränkungen in der elterlichen Feinfühligkeit kommen, was im Sinne eines Teufelskreises die kindliche Symptomatik weiter verstärkt [6]. Dennoch wurde diese theoretische Annahme bislang empirisch nicht hinreichend überprüft. Ein weiterer Aspekt, der häufig als ätiologischer Faktor frühkindlicher Regulationsprobleme diskutiert wird, ist eine psychische Erkrankung der Eltern. Querschnittstudien konnten zeigen, dass Eltern von Säuglingen mit Regulationsproblemen signifikant höhere Psychopathologiewerte aufweisen [23]. Eine Übersichtsarbeit, in die 30 Studien einbezogen wurden, zeigte, dass mütterliche Depression vielmehr Folge als Ursache exzessiven Schreiens ist, wohingegen eine Angstsymptomatik der Mutter sich als potenzieller Bedingungsfaktor für exzessives Schreien herausstellte. Mütterliche Depression scheint also – entgegen der landläufigen Meinung – keine Ursache für exzessives Schreien zu sein [24, 25].

In Bezug auf ätiologische Faktoren aufseiten des Kindes gibt es Hinweise, dass ein niedriges Geburtsgewicht und/oder ein geringeres Gestationsalter (speziell SGA^5^) die Wahrscheinlichkeit für Regulationsprobleme erhöhen kann [19, 26]. Darüber hinaus konnte gezeigt werden, dass Kinder mit einem schwierigen Temperament (z. B. niedrige Selbstregulationsfähigkeit, hoher negativer Affekt) häufiger Regulationsprobleme haben als Kinder mit einem einfachen Temperament. Insbesondere das Temperamentsmerkmal „negative Emotionalität“ scheint in Zusammenhang sowohl mit exzessivem Schreien [27], Fütterproblemen [28] als auch besonders mit Schlafproblemen [29] zu stehen. Psychosoziale Faktoren nehmen jedoch eine wichtige moderierende Rolle ein: Hagekull et al. konnten beispielsweise einen Interaktionseffekt zwischen einem schwierigen kindlichen Temperament und mütterlicher Feinfühligkeit in Bezug auf Fütterprobleme identifizieren, was einen Hinweis auf die Interaktion von biologischen und psychosozialen Faktoren gibt [30]. Insgesamt besteht in Bezug auf die Ätiologie frühkindlicher Regulationsstörungen noch großer Forschungsbedarf.

## Verläufe frühkindlicher Regulationsstörungen

Frühkindliche psychische Störungen können nur auf das Säuglingsalter beschränkt sein und in den ersten Lebensjahren remittieren. Bei 20–50 % der Säuglinge persistieren die Regulationsprobleme jedoch bis ins Vorschulalter [31, 32]. Neben homotypischer Kontinuität findet sich auch häufig eine heterotypische Kontinuität, d. h., dass bei Säuglingen mit Regulationsproblemen eine erhöhte Wahrscheinlichkeit besteht, später andere psychische Symptome in Form von externalisierendem (z. B. Aggression) oder internalisierendem (z. B. Angst, Depression) Problemverhalten zu entwickeln [33, 34]. Kinder mit Schlafstörungen in den ersten Lebensjahren haben beispielsweise eine erhöhte Vulnerabilität für Angst und depressive Symptome im mittleren Kindesalter.

Der wohl prominenteste Befund ist der prädiktive Zusammenhang zwischen persistierenden und multiplen frühkindlichen Regulationsproblemen und dem Auftreten von Aufmerksamkeitsstörungen im Kindesalter [35, 36]. In einer großen Längsschnittstudie wurde gefunden, dass Regulationsprobleme in der frühen Kindheit mit höheren Raten an Problemverhalten in der mittleren Kindheit assoziiert waren, die wiederum prädiktiv für die Entwicklung von Symptomen einer Borderline-Persönlichkeitsstörung und depressiven Symptomen im Jugendalter waren [37]. In einer aktuellen Metaanalyse lag die kumulative Inzidenz für spätere Verhaltensprobleme bei Kindern mit frühkindlichen Regulationsproblemen bei 23,3 % (vs. 6,7 % in einer gesunden Kontrollgruppe), wobei im Falle multipler Regulationsprobleme die Verhaltensprobleme besonders ausgeprägt waren [38]. Probleme in den Bereichen exzessives Schreien, Schlafen und Essen können also den Startpunkt für negative Entwicklungsverläufe darstellen.

Ob ein Säugling mit einer frühkindlichen Regulationsstörung später eine psychische Störung entwickelt, ist von verschiedenen Risiko- und Schutzfaktoren abhängig, wobei hier biologische und psychosoziale Faktoren zusammenwirken [39]. Als wohl bedeutsamster Risiko- bzw. Schutzfaktor konnte die Qualität der Eltern-Kind-Interaktion identifiziert werden: Eine Längsschnittstudie kam beispielsweise zu dem Schluss, dass die Qualität der Eltern-Kind-Interaktion den Zusammenhang zwischen frühkindlichen Regulationsproblemen und späteren Verhaltensstörungen erklärt [40]. Dies bedeutet, dass negative Entwicklungsverläufe frühkindlicher Regulationsprobleme von der Qualität der Eltern-Kind-Interaktion abhängig sind. Ein weiterer Risikofaktor für einen negativen Verlauf frühkindlicher Störungen ist eine psychische Erkrankung eines Elternteils [41], unter anderem aus dem Grund, da diese häufig mit einer niedrigeren elterlichen emotionalen Verfügbarkeit assoziiert ist [42]. Ist die Mutter trotz psychischer Erkrankung ausreichend feinfühlig, kann dies vor negativen Entwicklungsverläufen schützen [43, 44].

Als Risikofaktor für negative Verläufe aufseiten des Kindes konnte ein schwieriges Temperament identifiziert werden: Kinder mit Regulationsstörungen, die zusätzlich ein schwieriges Temperament haben, weisen ein besonders erhöhtes Risiko auf, im Kindesalter Verhaltensprobleme zu entwickeln [34, 45]. Neuere Studien beschäftigen sich mit dem Einfluss (epi)genetischer Faktoren auf den Verlauf psychischer Erkrankungen. In Bezug auf die Entwicklung von Symptomen einer Aufmerksamkeitsdefizit‑/Hyperaktivitätsstörung (ADHS) bei 2–15 Jahre alten Kindern/Jugendlichen konnte ein Interaktionseffekt zwischen einem Polymorphismus im DRD4-Gen und Regulationsproblemen im Säuglingsalter gefunden werden [46]. Darüber hinaus fungierte ein DRD4-Polymorphismus als Risikofaktor für Essstörungen im Alter von 3 Jahren [47]. Träger des Allels DRD4-7r mit frühkindlichen Regulationsproblemen zeigen nur dann ein höheres Ausmaß an späteren Verhaltensproblemen, wenn sie im Säuglingsalter weniger mütterliche Responsivität erlebt hatten [48]. In Bezug auf den Verlauf frühkindlicher Regulationsstörungen scheinen also biologische und psychosoziale Faktoren zusammenzuwirken, wobei die genauen Wirkmechanismen weiterer Forschung bedürfen.

## Interventionen

In den letzten Jahrzehnten sind weltweit zahlreiche Behandlungs- und Interventionsansätze entstanden, die auf Säuglinge und Kleinkinder mit Regulationsstörungen ausgerichtet sind. Hier unterscheidet man zwischen Ansätzen, die auf eine direkte Verbesserung der Eltern-Kind-Interaktion abzielen und psychodynamischen Verfahren, die auf der Repräsentationsebene der Eltern ansetzen [49].

Vor über 30 Jahren entwickelte Mechthild Papoušek als Pionierin in Deutschland ein integratives, entwicklungsdynamisches, systemisches Konzept einer Eltern-Säuglings‑/Kleinkind-Beratung und -Psychotherapie, das sogenannte Münchner Modell [50], welches im Arbeitsumfeld der Autor:innen verwendet und im Folgenden beschrieben wird.

Zu Beginn der Beratung und Therapie steht eine interdisziplinäre diagnostische Analyse des Störungsbildes und seiner Entstehungsbedingungen. In einer ausführlichen medizinisch-psychologischen Anamnese werden sowohl kindliche Faktoren als auch elterliche Belastungen und Ressourcen erhoben. Die Eltern-Kind-Kommunikation wird in unterschiedlichen Kontexten (z. B. Füttern, Spiel, Wickeln) beobachtet und analysiert. Eventuelle organische Faktoren werden in einer fundierten neuropädiatrischen Untersuchung diagnostiziert.

Gemeinsam mit den Eltern werden Behandlungsziele erarbeitet, abgestimmt auf die jeweilige kindliche regulatorische, kognitive und emotionale Entwicklung sowie die Ressourcen und Belastungen innerhalb des psychosozialen Kontextes. Die Beratung und Therapie der Familien verfolgt dabei vor dem Hintergrund der Symptomtrias (Abb. 1) Behandlungsziele auf 3 Ebenen: die Besserung der kindlichen Symptomatik, die Unterstützung und Entlastung der zumeist psychisch und physisch massiv erschöpften Eltern und die Ermöglichung positiver Interaktions- und Beziehungserfahrungen zwischen Eltern und Kind.

Die wertschätzende und haltgebende therapeutische Grundhaltung ermöglicht es den verunsicherten und hochbelasteten Familien, sich hinsichtlich ihrer Sorgen und problematischeren Gefühle zu öffnen. Der sichere und validierende Kontext bietet den Eltern zudem die Möglichkeit, korrigierende Beziehungserfahrungen zu erleben und Hoffnung auf Besserung der aktuellen Situation zu entwickeln [5, 8].

Schwangerschaft, Geburt und die ersten Lebensjahre sind sowohl für den Säugling als auch für die Eltern eine Phase rasanter Entwicklungsdynamik mit weitreichenden Veränderungen und Anpassungsprozessen. Beim Übergang zur Elternschaft durchleben die Eltern tiefgreifende Reorganisationsprozesse, die neue Bedürfnisse, Fantasien und Ängste hervorrufen können. In dieser vulnerablen Umbruchsituation werden eigene frühe bewusste und unbewusste Beziehungserfahrungen reaktiviert und neu belebt [8].

**Figure 2:**
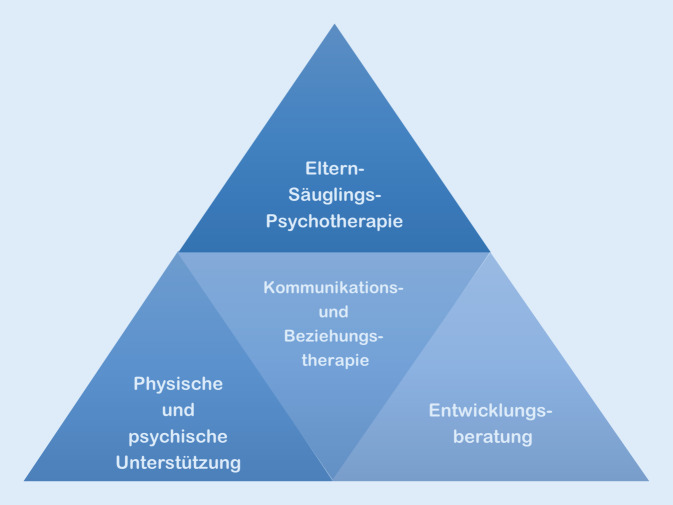

Das therapeutische Konzept versucht, dieser besonderen psychobiologischen Entwicklungsphase gerecht zu werden und umfasst 3 zentrale Bausteine: Entwicklungsberatung, physische und psychische Unterstützung der Eltern sowie Kommunikations- und Beziehungstherapie. Bei Bedarf werden sie durch Eltern-Säuglings-Psychotherapie ergänzt (Abb. 2). In welchem Maße die einzelnen Elemente zur Anwendung kommen, wird durch Symptomatik, die psychosoziale Situation und die Bedürfnisse der Familie bestimmt [2].

### Entwicklungsberatung

Hier werden den Familien entwicklungspsychologische Informationen, wie z. B. Regulationsfähigkeit, Schlafbedarf, Nahrungsaufnahme, Trennungsängste, Autonomieentwicklung und die individuelle Variabilität der kindlichen Entwicklung, vermittelt. Dies ermöglicht den Eltern, ihr Kind mit seinem Temperament, seinen entwicklungsbedingten Fähigkeiten und seinen Stärken und Schwächen wahrzunehmen und die Welt aus der Sicht des Kindes zu sehen. Die sich daraus entwickelnden reflexiven Fähigkeiten ermöglichen den Eltern, ihren Säugling affektiv und kognitiv zu verstehen und sich in seine Erlebenswelt einzufühlen. Ein hohes elterliches Reflexionsniveau stellt zudem einen Schutzfaktor für die weitere Entwicklung des Kindes dar [51].

### Physische und psychische Unterstützung der Eltern

In ressourcenorientierten, psychotherapeutischen Gesprächen bekommen die Familien Raum für ihre aktuellen Belastungen, Verunsicherungen und oft schambesetzten Gedanken und Emotionen. In einer haltgebenden und wertschätzenden therapeutischen Beziehung können sich die Eltern mit belastenden Gefühlen, wie zum Beispiel Enttäuschung, Trauer, Wut und Verzweiflung, öffnen. Neben der psychischen Belastung sind die Eltern exzessiv schreiender oder schlafgestörter Säuglinge zumeist äußerst erschöpft und leiden unter einem massiven Schlafdefizit. Hier suchen wir nach familiärer oder institutioneller Unterstützung [2].

### Kommunikations- und Beziehungstherapie

Ein zentraler Bestandteil der Behandlung liegt in der Kommunikations- und Beziehungstherapie. Diese kann mithilfe von videografierten Interaktionssequenzen oder direkt anhand der spontanen Eltern-Kind-Interaktionen in den Therapiestunden erfolgen. Ziel dabei ist es, die Eltern für die Signale des Kindes zu sensibilisieren und Momente positiver Gegenseitigkeit (sog. Engelskreise) zu erleben und zu genießen. Diese positiven Beziehungsmomente bilden ein Gegengewicht zu den belastenden und wiederkehrenden Teufelskreisen. Im geschützten therapeutischen Rahmen können den Eltern Beziehungserfahrungen ermöglicht werden, in denen sie ihren Säugling intuitiv unterstützen und dadurch Vertrauen in ihre Kompetenz als Eltern entwickeln können [2].

In vielen Fällen helfen diese basalen Elemente, um eine rasche Besserung der Grundsymptomatik des Kindes sowie der familiären Belastung zu erreichen. Bei besonderer Schwere der kindlichen Symptomatik, massiver elterlicher Belastung bei fehlendem psychosozialen Netz, psychischen Erkrankungen der Eltern oder schwerer Kommunikations- und Beziehungsstörung kommt die Eltern-Säuglings‑/Kleinkind-Psychotherapie zum Einsatz.

### Eltern-Säuglings‑/Kleinkind-Psychotherapie

Wenn Eltern ihre Kinder verzerrt wahrnehmen, ihre eigenen, durch die Säuglinge hervorgerufenen Affekte nicht regulieren können, sie diese dem Kind ungefiltert zumuten und die intuitiven Kompetenzen massiv beeinträchtigt sind, ist eine psychotherapeutische Begleitung indiziert. Säuglinge und Kleinkinder, die in diesem Spannungsfeld aufwachsen, sind in ihrer biopsychosozialen Entwicklung massiv gefährdet. In engmaschigen therapeutischen Sitzungen wird mit den Eltern an der Bewusstmachung der projektiven Verzerrung der Wahrnehmung ihres Kindes und der Zusammenhänge mit eigenen belastenden und traumatischen frühen Erfahrungen gearbeitet [5, 49].

Ein Beispiel wäre, wenn die Mutter in ihrem 2 Monate alten, exzessiv schreienden Säugling Aspekte ihres cholerischen Vaters wahrnimmt und mit Panik oder verzweifelter Wut auf ihn reagiert. Sie kann, aufgrund der Reaktivierung ihrer eigenen traumatischen Geschichte, in diesen Momenten ihr Baby nicht als hilflos, überreizt und belastet wahrnehmen, sondern vielmehr als bedrohlichen Aggressor. Sie selbst fühlt sich in diesen Momenten als kleines, bedrohtes Mädchen, welches seinem Vater hilflos ausgesetzt ist. Sie kann in diesen Momenten ihre Rolle als Mutter nicht einnehmen. Der Säugling, der in Phasen von Überreizung und Belastung die co-regulatorische Unterstützung seiner Mutter dringend bräuchte, sieht sich nun aggressiven Impulsen ausgeliefert oder alleingelassen. Ein Teufelskreis entsteht, welcher unbehandelt mit Störungen der Affektregulation und der Bindungs- und Beziehungsentwicklung assoziiert ist.

Die Behandlung von frühkindlichen Störungsbildern erfolgt in enger interdisziplinärer Kooperation mit Pädiater:innen, Psychotherapeut:innen, Kinder- und Jugend- sowie Erwachsenenpsychiater:innen, ergo-, physio- oder weiteren fachtherapeutischen Kolleg:innen, den Frühen Hilfen und der Jugendhilfe. Wenn die ambulanten Hilfsangebote an ihre Grenzen kommen, müssen teilstationäre oder stationäre Behandlungsmaßnahmen in Betracht gezogen werden [7].

Die Wirksamkeit von unterschiedlichen Formen der Eltern-Säuglings‑/Kleinkind-Psychotherapien wurde in Evaluationsstudien mehrfach aufgezeigt. Es zeigen sich sowohl Effekte in der Reduktion der kindlichen Verhaltensprobleme und der elterlichen Belastung als auch eine Verbesserung der Qualität der Eltern-Kind-Interaktion und -Beziehung [2, 52, 53]. Diese Effekte sind stabil bis zu 6 Monate nach Behandlungsende nachweisbar [2, 52].

## Frühkindliche Störungen im Kontext der COVID-19-Pandemie

Die COVID-19-Pandemie stellte insbesondere für viele Familien mit Säuglingen und Kleinkindern eine psychosoziale Belastung dar, deren Langzeitfolgen noch nicht absehbar sind und aktuell in diversen Studien untersucht werden. Eine Metaanalyse über 18 Studien aus den Jahren 2021 und 2022 liefert Hinweise auf eine Prävalenz von 27,4 % klinisch relevanter depressiver Symptome sowie 43,4 % klinisch erhöhter Angstwerte bei Müttern von Kindern jünger als 5 Jahre während der Pandemie [54, 55] sowie auf eine stärkere psychische Belastung in der Peripartalzeit [56].

Vor allem in psychosozial vorbelasteten Familien und bei alleinerziehenden Elternteilen scheint das Risiko für psychische Belastungen im Alltag verstärkt zu sein [57]. Mütter von Babys, welche während der COVID-19-Pandemie geboren wurden, berichteten von höherer eigener Unzufriedenheit nach der Geburt des Kindes und von vermehrtem Auftreten von exzessivem Schreien und Schlafstörungen bei ihren 7 Monate alten Babys [58]. In einer anonymisierten Onlinebefragung in deutschsprachigen Ländern (D-A-CH) während des ersten Lockdowns 2020 wurden 5823 Personen mit Kindern bzgl. der eigenen Belastungen und der Verhaltensauffälligkeiten ihrer Kinder befragt. Deutlich wurde eine signifikante Zunahme von Affektregulationsstörungen und dysregulierten Verhaltensweisen bei Kindern im Alter von 0–6 Jahren, insbesondere wenn die psychische Gesundheit ihrer Eltern beeinträchtigt war [59]. Einschränkend muss erwähnt werden, dass es ich bei den Studien überwiegend um Querschnittsanalysen handelt und von daher noch keine Rückschlüsse auf die längerfristigen Auswirkungen gezogen werden können. Dazu wären weitere Verlaufsuntersuchungen notwendig.

Säuglinge und ihre Eltern verfügen in der Regel über genügend Ressourcen vorübergehende Belastungen gut zu überstehen. Bei persistierenden psychosozialen und familiären Belastungen sowie einer belasteten Eltern-Kind-Beziehung ist die sozioemotionale und weitere psychische Entwicklung der Säuglinge und Kleinkinder massiv gefährdet. Für diese Familien werden dringend niederschwellige Unterstützungssysteme sowie kompetente Beratungs- und Therapieangebote benötigt.

## Fazit

Zusammenfassend lässt sich festhalten, dass es zu wenig empirische Evidenz bezüglich spezifischer ätiologischer Faktoren von frühkindlichen psychischen Störungen gibt, wobei bezüglich der Aufrechterhaltung der Störung von einem Zusammenwirken biologischer und psychosozialer Faktoren (= biopsychosoziales Modell) ausgegangen werden kann. Insgesamt gibt es ausreichend Evidenz dafür, dass Säuglinge mit multiplen und/oder persistierenden Regulationsstörungen, wie exzessives Schreien über den 6. Lebensmonat hinaus, ein erhöhtes Risiko für psychische Erkrankungen auch im Langzeitverlauf haben. Um negativen Entwicklungsverläufen entgegenzuwirken, sind frühzeitige Interventionen angezeigt, die an den aus der Forschung bekannten Schutzfaktoren (u. a. Stärkung der Eltern-Kind-Beziehung) ansetzen.

## Footnotes

1 AWMF: Arbeitsgemeinschaft der Wissenschaftlichen Medizinischen Fachgesellschaften.

2 ICD: Internationale statistische Klassifikation der Krankheiten und verwandter Gesundheitsprobleme.

3 DSM: Diagnostischer und statistischer Leitfaden psychischer Störungen.

4 DC:0–5: Diagnostic Classification of Mental Health and Developmental Disorders of Infancy and Early Childhood.

5 SGA: „small for gestional age“, bezeichnet ein zu geringes Gewicht bezogen auf das Gestationsalter (Anzahl der Wochen zwischen dem ersten Tag der letzten normalen Menstruation der Mutter und dem Tag der Entbindung).
